# Sexual selection on male but not female function in monoecious and dioecious populations of broadleaf arrowhead (*Sagittaria latifolia*)

**DOI:** 10.1098/rspb.2022.0919

**Published:** 2022-11-09

**Authors:** Allison Kwok, Marcel E. Dorken

**Affiliations:** ^1^ Environmental and Life Sciences Graduate Program, Trent University, Peterborough, ON, Canada K9J 0G2; ^2^ Department of Biology, Trent University, Peterborough, ON, Canada K9J 0G2

**Keywords:** Bateman gradients, clonal growth, dioecy, modularity, monoecy, sexual systems

## Abstract

Direct measures of sexual selection in plants are rare and complicated by immobility and modular growth. For plants, instantaneous measures of fitness typically scale with size, but covariances between size and mating success could obscure the detection of sexual selection. We measured the magnitude of sexual selection in a monoecious and a dioecious population of the clonal plant *Sagittaria latifolia* using Bateman gradients (*ß_ss_*). These gradients were calculated using parentage analysis and residual regression to account for the effects of shoot and clone size on mating and reproductive success. In both populations, (i) there was greater promiscuity via male function than via female function and (ii) *ß_ss_* were positive, with significant associations between mating and reproductive success for male but not female function. Moreover, estimated *β_ss_* were similar for the monoecious and dioecious populations, possibly because non-overlapping female and male sex phases in hermaphroditic *S. latifolia* reduced the scope for interference between sex functions during mating. This study builds on previous studies of selection on plant mating traits, and of sexual selection under experimental conditions, by showing that sexual selection can operate in natural populations of plants, including populations of hermaphrodites.

## 1. Introduction

Sexual selection arising from fitness differences in reproductive success via non-random variation in mating success is a key process affecting organismal evolution in anisogamous lineages, including animals [1] and plants [2,3]. Mating regulates the transmission of genes from one generation to the next, so sexual selection can have important consequences not only for individual fitness, but also for patterns of genetic diversity within populations [3,4], reproductive isolation [5] and speciation [6,7]. Several lines of evidence point to the importance of sexual selection in shaping patterns of sexual dimorphism in dioecious plants (i.e. populations of distinct female and male individuals [8]), variation in floral traits [9–11] and the processes regulating pollination [12] and fertilization success [9,10,13–15]. However, very few studies of sexual selection in plants have included measures of mating success (i.e. number of mates [15,16]). As a result, studies directly quantifying the strength of sexual selection in plants remain rare [2,16].

The measurement of sexual selection in plants has lagged studies of animals, at least in part because the occurrence of sexual selection in plants has been contentious [2]. Mate choice is an important driver of sexual selection [17–19] and plants, which rely on third-party vectors for pollination, lack the ability to directly choose mates during pollination [2,20,21]. However, sexual selection via mate choice does not require the active choosing of mates [22], and several aspects of plant reproduction can yield sexual selection [2], including male–male competition in sporophytic (during pollination) [23] and gametophytic (post-pollination) phases of the life cycle [23–25]. Indeed, there may even be scope for female choice in plants via sporophyte–gametophyte interactions [3]. Nevertheless, sexual selection may be a weaker force in hermaphroditic than in dioecious populations if there is reciprocal mating and/or male and female sex functions are expressed simultaneously [26]. These long-standing issues notwithstanding, sexual selection is now widely considered to be an important evolutionary force in plants, even if the magnitude of sexual selection may be weaker in hermaphroditic compared to dioecious populations.

An additional and less widely discussed problem with the measurement of sexual selection in plants is that measures of fitness usually scale with plant size. Although individual size is considered a sexually selected trait for some unitary organisms [27–29], size variation poses a challenge to the inference of sexual selection for modular organisms like plants. For plants, as for some unitary organisms [30,31], size varies with age and reflects ongoing trade-offs in the allocation of resources to growth versus survival and reproduction [32,33]. For unitary animals with indeterminate growth, these trade-offs make size a sensible trait when considering the operation of sexual selection (i.e. there may be heritable variation in size among individuals within age cohorts [34]). Although (non-heritable) variation in local resource conditions should contribute to variation in size for plants and animals, immobility further exacerbates these effects for plants. Moreover, modularity and opportunities for independent resource capture among modules might weaken trade-offs between growth and reproduction. Variation in plant size may therefore obscure patterns of sexual selection whenever plant size covaries with reproductive and mating success. In particular, underlying covariances between plant size and reproductive and mating success should yield positive measures of the strength of sexual selection even under random mating—in this scenario, some phenotypes (large plants) can be expected to have high reproductive and mating success, while other phenotypes (small plants) have low reproductive and mating success. Accordingly, rather than considering plant size a sexually selected trait, studies of sexual selection in plant populations control for variation in size among individuals (e.g. by including size as a covariate when estimating the strength of sexual selection [35], or via the use of standardized growth conditions for same-aged plants [16]).

A variety of metrics for estimating the strength of sexual selection exist, but they vary in their utility [36,37]. For example, one of the most widely used metrics of sexual selection, *I*_s_ (the opportunity for sexual selection), measures the variance in mating success but, among other problems [37], cannot be scaled to take variation in plant size into account. Selection gradients and/or selection differentials are useful when specific traits are hypothesized to be subject to sexual selection [37]. But by being trait-specific these metrics do not estimate the total strength of sexual selection within populations [38]. Bateman gradients are one of the most direct methods for quantifying the overall strength of sexual selection [26,38]. They are calculated from the slope of the association between mating success and reproductive success for each sex function, and under Bateman's third principle (the relationship between mating and reproductive success is stronger for males than for females [39]), are expected to be steeper for male than for female function [40]. Bateman gradients have been used to examine the strength of sexual selection in a range of animal groups [26,40,41], and more recently in plants [16,41].

Here, we investigated patterns of sexual selection in natural populations of the clonal aquatic plant *Sagittaria latifolia*. Populations of *S. latifolia* may be either monoecious (populations of hermaphroditic plants with separate female and male flowers) or dioecious. Previous studies of this plant have indicated that reproductive traits, such as male floral display size, might be subject to selection [42–44], including sexual selection [23]. Moreover, the duration of flowering and number of flowers produced vary between the sexes (or between sex functions in monoecious populations) in a manner that might affect the magnitude of sexual selection. Using direct measures of reproductive success and indirect measures of mating success determined using parentage analysis from the segregation of simple-sequence repeat (SSR) alleles, we estimated the degree of promiscuity, the opportunity for sexual selection and the magnitude of Bateman gradients for females and males in a dioecious population, and for female and male function in a monoecious population. We expected mating and reproductive success to be strongly size-dependent for *S. latifolia* [46]. Accordingly, we adjusted our primary measure of sexual selection (the Bateman gradients) for variation in plant size and compared this size-adjusted measure to unadjusted measures that were based purely on patterns of mating and reproductive success (*I*_s_ and an index of promiscuity).

## 2. Methods

### (a) Study species

*Sagittaria latifolia* Willd. is an emergent clonal aquatic plant, commonly found in wetland habitats across North America. Shoots (ramets) are produced from axillary stolons during the growing season and can be vegetative or reproductive. Corms are also produced from axillary stolons and function as perennating structures [45]. Genetic individuals (genets) may be composed of multiple ramets that are temporarily interconnected by stolons, which, along with all above-ground vegetative tissues, deteriorate at the end of each growing season. Variation in plant size is driven by clonal expansion of genets [46,47] and by variation in ramet size that is primarily determined by local growing conditions [45].

Flowers are produced on racemes with three flowers at each node. In monoecious populations, female flowers occupy basal nodes and male flowers occupy distal nodes (electronic supplementary material, figure S1). Flowers open basipetally, remain open for a single day, and nodes with male flowers mature later than nodes with female flowers, resulting in inflorescences that are synchronously protogynous (i.e. there is usually no overlap between female and male sex phases [47]). In dioecious populations, sex is genetically determined and ramets produce male or female flowers [48]. For both monoecious and dioecious ramets, female flowers tend to open simultaneously, while male flowers open sequentially, usually one whorl at a time. In Southern Ontario, flowering occurs from late June through to mid-September and flowers are pollinated by a variety of insects [42]. Seed set is not pollen-limited in monoecious populations [43]. In both monoecious and dioecious populations, male flowers have larger petals than female flowers, but female floral displays are larger due to greater floral synchrony [44]. In dioecious populations, the number of flowers produced per ramet is size-dependent for both sexes [44,49]. In monoecious populations, only female flower production is size-dependent [44,49]. Plants are self-compatible, but due to synchronous protogyny, self-fertilization within ramets is unlikely [50]. Selfing between flowering ramets of the same genet can occur. However, as clones increase in size, they tend to intermingle with ramets from unrelated genets such that selfing rates—at least in the monoecious population studied here—may be only weakly associated with variation in genet (clone) size [46].

### (b) Study site and floral sampling

To survey flowering in *S. latifolia*, we sampled a monoecious and a dioecious population for each of five consecutive days. The monoecious population was located in a shallow area of Thompson Creek in Meadowvale Park, Peterborough, ON, Canada (44° 20′ N, 78° 18′ W). Flowering shoots at this site were sampled from 8 to 12 August 2016 in a 13 × 17 m area of the population. The dioecious population occupied a 13 × 4 m area of a shallow roadside ditch beside an agricultural field near Stoney Point, ON (44° 17′ N, 82° 28′ W). Plants at this site were sampled between 21 and 26 August 2016. Due to rain and the absence of pollinators, there was no sampling on 23 August. On the first day of sampling at each site, study zones were delimited to areas of approximately 100 flowering ramets and open flowers were marked with tags. At the monoecious site, the study zone was separated from the rest of the population by natural gaps between patches of *S. latifolia* and ramets outside the study zone were prevented from mating with plants in the study zone by removing all male flowers on plants outside the zone. At the dioecious site, all flowering ramets in the population were included in the study.

Within each population, open female flowers were tagged and labelled each day to enable tracking of the date of pollination. The numbers of open female and male flowers were recorded each day for all flowering ramets in the study zone. The mean numbers of flowering ramets per day were similar for the monoecious (71.60 ± 15.14 s.d. flowering ramets per day) and dioecious populations (68.80 ± 22.54 s.d.). A total of 167 flowering ramets were tracked at the monoecious site, and 183 flowering ramets were tracked at the dioecious site.

Developing fruits were collected approximately one month after initial sampling. The one-month gap between floral sampling and fruit collection resulted in numerous fruits not being recovered due to disturbances from flooding and foraging by wildlife. Because of these disturbances, the total sample of fruits obtained was a subset of the total number of ramets with female flowers in each population (33/113 in the monoecious population; 38/54 in the dioecious population). Once collected, fruits were allowed to dry at room temperature and then stored in air-tight containers at 4°C. Fruits were stored individually in coin envelopes and labelled with the ramet ID and flowering date. Prior to DNA extraction, a random subset of the seeds from each fruit were soaked in water to enable the removal of maternally derived tissues around the embryo. A total of nine seeds per fruit per ramet were subsequently sampled for genotyping and parentage analysis.

### (c) Genet and paternity assignment

Leaf and seed DNA were extracted and genotyped using microsatellite loci following procedures outlined in the electronic supplementary material (electronic supplementary material, DNA extraction and SSR genotyping; and see [46]). Ramets were assigned to genets (here defined as multi-locus lineages, MLLs) (electronic supplementary material, Genet and paternity assignments). Ramets with multi-locus genotypes (MLGs) that differed by one allele were assigned to the same MLL to avoid subdividing genets with slightly different MLG scores because of potential genotyping errors or somatic mutations [51]. Seeds were assigned paternity using software that groups parents and offspring into paternal and maternal sibships by maximizing the likelihood of the probability of these sibships based on their MLGs [52–54] The paternal parent of each seed was assumed to be the MLL with the greatest probability of paternity (as estimated by COLONY2 [53], the probability of paternity is indicated as *π* in the calculations below), and calculations of the reproductive success via male function (paternity share) for each MLL were weighted by *π*. Further details on genet and paternity assignments are provided in the electronic supplementary material.

### (d) Reproductive and mating success

Reproductive success via female function (*RS*^f^) was estimated as the total number of seeds produced per MLL *i*:

$$RS_{i}^{f}=\underset{\;j\;}{\sum }\underset{k}{\sum }N_{\;jk}^{S},$$

where *N*^S^ was the estimated number of seeds produced per pollination date *k* by each ramet *j* within MLL *i*. Because ramets typically produce thousands of seeds, we estimated *N*^S^ as the quotient of the total mass of all fruits per ramet and the average mass of individual seeds (achenes).

Paternity share (*RS*^m^) was calculated as the estimated number of outcrossed seeds sired per MLL *i*:

$$RS_{i}^{m}=\underset{j}{\sum }\underset{k}{\sum }\left[\left(\frac{\sum_{l}\pi_{ijkl}}{N_{\;jk}^{G}}\right)\times N_{\;jk}^{S}\right],$$

where *π_ijkl_* is the probability that each genotyped seed *l* per maternal ramet *j* was sired by MLL *i* on pollination date *k*, *N*^S^ is the estimated total number of seeds per maternal ramet and *N*^G^ is the total number of seeds genotyped per maternal ramet. Self-fertilized seeds were excluded from calculations of mating and reproductive success. There is substantial inbreeding depression in monoecious populations of *S. latifolia* [50], reducing the expected contribution of selfed offspring to reproductive success.

We estimated the genetic mating success for female and male function in each population as the total number of mates obtained per MLL via each sex function, as identified from parentage analysis. We refer to these estimates as proxies of mating success because we inferred mating events from the products of fertilization rather than from direct observation. For female function, the proxy of mating success in each population (*MS*^f^) was calculated as the number of unique sire MLLs (*N*^m^) with seeds on maternal MLL *i* summed across its *j* ramets:

$$MS_{i}^{f}=\underset{j}{\sum }N_{j}^{m}.$$

Male mating success (*MS*^m^) was estimated by summing the number of unique maternal MLLs (*N*^f^) with seeds sired by each MLL *i* across its *j* ramets as follows:

$$MS_{i}^{m}=\underset{j}{\sum }N_{j}^{f}.$$

For the calculation of Bateman gradients, mating success and reproductive success were each mean standardized [16,18].

To quantify promiscuity, we calculated an index of mate diversity using the parentage assignments of our sample of genotyped seeds. Mate diversity (*M*) was calculated for each sex (or sex function) as the number of mating partners per MLL *i* (*MS_i_*), divided by *e*, the total number of seeds for which an MLL was assigned as the parent (i.e. the known maternal parent or the inferred paternal parent):

$$M_{i}=\frac{MS_{i}-1}{e_{i}-1}.$$

We subtracted one from both the numerator and the denominator so that the index varied between 0 and 1, where a value of 1 indicated that all mating events involved different MLLs. The minimum value of 0 was not possible in this study—we only tracked mating and reproductive success for MLLs that produced seeds and/or were involved in one or more siring events.

### (e) Quantifying sexual selection

To estimate the magnitude of sexual selection in each population, we calculated (i) the opportunity of selection (*I*) – the relative variance in reproductive success; (ii) the opportunity for sexual selection (*I*_s_) – the relative variance in mating success and (iii) the Bateman gradient (*β*_ss_) – the slope of the association between mating and reproductive success (electronic supplementary material, Bateman gradients—calculated using residual regression). Both *I* and *I*_s_ are standardized and unitless, enabling comparisons between sexes, and across studies [38]. However, *I* and *I*_s_ cannot be calculated in a way that accounts for size effects—if there are underlying associations between size and reproductive and mating success, values of *I* and *I*_s_ might be subject to a positive bias if there is variation in plant size. Moreover, these two measures can—at least in principle—provide misleading inferences regarding the strength of sexual selection [36] and are most useful when reported alongside other measures of the strength of sexual selection [18,37,38,55].

We estimated *I* and *I*_s_ by first dividing the variance of reproductive and mating success by their respective squared means for each sex (for the dioecious population: *I*_m_ and *I*_f_, and *I*_sm_ and *I*_sf_) or sex function (for the monoecious population: *I*_mm_ and *I*_ff_, and *I*_smm_ and *I*_sff_). Binomial errors were calculated for *I*_s_ to account for an extra binomial error that can arise from sampling effects [40]. Sex differences in the opportunity of selection (*I*) and sexual selection (*I*_s_) were calculated as *I*_m_ ∓ *I*_f_ and *I*_sm_ – *I*_sf_ for the dioecious population; *I*_mm_ – *I*_ff_ and *I*_smm_ – *I*_sff_ for the monoecious population [18].

Bateman gradients were calculated using residual regression to control for the effects of ramet and genet size. We first calculated least-squares multiple regressions of mating and reproductive success as functions of genet size (number of flowering ramets per MLL) and ramet size (average mid-vein length per ramet per MLL). In these models, *relative mating success* or *relative reproductive success* were the dependent variables, and *ramet size* and *log_10_(genet size)* were the independent variables (genet size was log-transformed to account for right-skew in the data). Bateman gradients were then calculated separately for each sex (or sex function; electronic supplementary material, Bateman gradients calculated using residual regression) as the slope of the least-squares regression of standardized residual reproductive success on standardized residual mating success as follows:

$$\begin{array}{rl} RS_{\mathrm{resid}}^{f}= & \alpha +\beta_{f}MS_{\mathrm{resid}}^{f}+\varepsilon \\ RS_{\mathrm{resid}}^{m}= & \alpha +\beta_{m}MS_{\mathrm{resid}}^{m}+\varepsilon . \end{array}$$

The slope of each regression was then the sex-specific Bateman gradient (*β*_f_ and *β*_m_ for the dioecious population; *β*_ff_ and *β*_mm_ for the monoecious population) that describes how variation in mating success contributed to reproductive success and ε is an (non-constant) error term. For each population, the sex difference in sexual selection (Δ*β*_ss_) was calculated using separate least-squares multiple regression models that included a (residual) mating success × sex interaction term, where Δ*β*_ss_ corresponds to the slope coefficient for the interaction term. Combined with significant values of *β*_m_ or *β*_f_, values of Δ*β*_ss_ indicate whether sexual selection is stronger in one sex (or sex function) than the other [38]. All regression analyses were calculated using the lm function in R [56]. We present alternative analyses that accounted for variation in plant size (multiple regression [57]) or that involved subsampling of equal-sized MLLs via bootstrapping in the electronic supplementary material. Finally, we also estimated Bateman gradients without accounting for variation in plant size and contrast the findings with those from residual regression (electronic supplementary material—Bateman gradients uncorrected for plant size).

In monoecious populations, reproductive success via one sex function might be affected by mating events that involve the other sex function. For example, if the presence of female sex organs reduces pollen export, reproductive success via male function may be positively associated with mating success through male function but negatively associated with mating success via female function [58]. For this reason, cross-sex effects should be considered when estimating Bateman gradients in populations of hermaphrodites [26]. However, we recovered an insufficient number of hermaphrodites that were inferred to have been both sires and mares to calculate these cross-sex effects (*n* = 4 MLLs). Despite this, we expect these cross-sex effects to be minimal for monoecious *S. latifolia*—hermaphrodites in monoecious populations of *S. latifolia* are synchronously protogynous with unisexual flowers, meaning that at any point in time hermaphroditic ramets are functionally dioecious.

## 3. Results

### (a) Flowering and genet size

Out of the 140 MLLs in the monoecious population, the vast majority were small, with 90% comprising a single flowering ramet. The largest MLL was composed of 14 flowering ramets. In the dioecious population, where 110 MLLs were detected, genets were also skewed towards smaller sizes and 87% of MLLs were composed of a single flowering ramet. However, the largest MLLs were bigger in the dioecious populations than in the monoecious population, with one MLL having a total of 28 flowering ramets. There were 14 multi-ramet genets in both the monoecious and dioecious populations (electronic supplementary material, figure S2).

**Figure 1.**
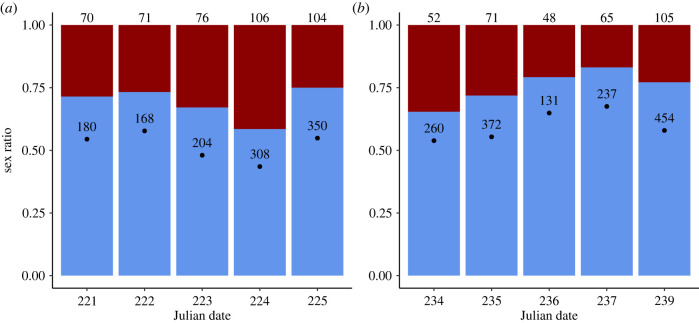
Daily ramet and floral sex ratios in a monoecious and a dioecious population of *Sagittaria latifolia*. Daily ramet sex ratios are indicated by height of the segments in each bar (lower segment, male; upper segment, female). (*a*) In the monoecious population, ramet sex ratios reflect the proportion of ramets with open male or open female flowers. (*b*) In the dioecious population, genet sex ratios reflect the proportion of male versus female ramets flowering on that day. Floral sex ratios (number of open male flowers divided by the total number of open flowers per day) are indicated by the location of the black circle within each bar. Numbers above each bar indicate the number of ramets with open flowers per day and numbers in the bars represent the number of open flowers per day. (Online version in colour.)

In the monoecious population, the average numbers of open female and male flowers per day were approximately equal (mean daily floral sex ratio = 0.52 ± 0.03 s.e., calculated as proportion of males/total number of male and female flowers; figure 1*a*), but in the dioecious population there were, on average, more open male flowers than female flowers (mean daily floral sex ratio = 0.60 ± 0.03 s.e.; figure 1*b*). In the monoecious population more ramets flowered in male-phase than in female-phase per day (mean daily proportion male-phase ramets = 0.69 ± 0.03 s.e., calculated as the proportion of male-phase ramets versus total flowering ramets per day; figure 1*a*). The same was true in the dioecious population where more male than female ramets flowered each day (mean daily proportion male ramets = 0.75 ± 0.03 s.e.; figure 1*b*).

### (b) Size effects on mating and reproduction

In both populations, larger genets produced more flowers (electronic supplementary material, table S2 and figure S3) and unsurprisingly, had greater mating and reproductive success (electronic supplementary material, table S6). In the monoecious population, genet size was positively associated with mating and reproductive success for both female and male function (multiple linear regression: female function *MS* = 0.32 + (1.49 × genet size) + (0.03 × ramet size), *F*_2,22_ = 7.30, *p* < 0.01, *R*^2^ = 0.34; female function *RS* = 0.10 + (4.01 × genet size) + (0.03 × ramet size), *F*_2,22_ = 26.20, *p* < 0.01, *R*^2^ = 0.68; male function *MS* = 0.95 + (7.36 × genet size) – (0.02 × ramet size), *F*_2,15_ = 78.21, *p* ≤ 0.001, *R*^2^ = 0.90; male function *RS* = 1.11 + (8.75 × genet size) – (0.03 × ramet size), *F*_2,15_ = 37.39, *p* < 0.001, *R*^2^ = 0.81; electronic supplementary material, table S6). Patterns were the same in the dioecious population, where the association between genet size and mating and reproductive success was positive for both sexes (multiple linear regression: female *MS* = 0.80 + (1.20 × genet size) + (0.003 × ramet size), *F*_2,13_ = 12.19, *p* < 0.01, *R*^2^ = 0.60; female *RS* = −0.41 + (3.80 × genet size) + (0.06 × ramet size), *F*_2,13_ = 142.10, *p* < 0.001, *R*^2^ = 0.95; male *MS* = −0.13 + (1.38 × genet size) + (0.06 × ramet size), *F*_2,6_ = 6.67, *p* = 0.03, *R*^2^ = 0.59; male *RS* = −0.05 + (1.15 × genet size) + (0.05 × ramet size), *F*_2,6_ = 4.50, *p* = 0.06, *R*^2^ = 0.47; electronic supplementary material, table S6).

### (c) Promiscuity and the opportunity for sexual selection

Mating was highly promiscuous in both populations, particularly via male function. In the monoecious population, MLLs had nearly twice the mate diversity via their male function (*M*_mm_ = 0.52 ± 0.09 s.e.) than via their female function (*M*_ff_ = 0.27 ± 0.03 s.e.). The same was true in the dioecious population, where males had an average mate diversity of *M*_m_ = 0.40 (± 0.05 s.e.), compared to *M*_f_ = 0.22 (± 0.03 s.e.) for females. High levels of promiscuity were associated with substantial variance in mating and reproductive success in both populations.

In the monoecious population, the variance in *RS* was more than three times greater for male function (*I*_mm_ = 3.12 ± 0.03 s.e.) than for female function (*I_ff_* = 0.93 ± 0.04 s.e.), yielding a greater opportunity for selection via male function (Δ*I* = 2.19; table 1). Similarly, the opportunity for sexual selection was more than seven times greater for male function *(I*_smm_ = 1.93 ± 0.03 s.e.) than for female function (*I*_sff_ = 0.26 ± 0.04 s.e.), leading to a positive value of Δ*I*_s_ (1.67; table 1). By contrast, in the dioecious population, the opportunity for selection was greater for females (*I*_f_ = 1.84 ± 0.09 s.e.) than for males (*I*_m_ = 0.57 ± 0.04 s.e.), yielding a negative value of Δ*I* (−1.27)*.* However, the opportunity for sexual selection was greater in males (*I*_sm_ = 0.69 ± 0.04 s.e.) than in females (*I*_sf_ = 0.26 ± 0.09 s.e.), leading to a positive value of Δ*I*_s_ (0.43).

**Table 1.** Sexual selection metrics for female function MLLs (*n* = 25) and male function MLLs (*n* = 18) in a monoecious population and for female MLLs (*n* = 16) and male MLLs (*n* = 9) in a dioecious population of *Sagittaria latifolia*.

	*I* (s.e.)	*I*_s_ (s.e.)	*ß* (s.e.)
monoecious			
female function	0.93 (0.04)	0.26 (0.04)	0.42 (0.26)
male function	3.12 (0.03)	1.93 (0.03)	1.02 (0.35)
Δ (male – female)	2.19	1.67	0.60 (0.42)
dioecious			
female	1.84 (0.09)	0.26 (0.09)	0.20 (0.25)
male	0.57 (0.04)	0.69 (0.04)	0.95 (0.15)
Δ (male – female)	−1.27	0.43	0.76 (0.30)

### (d) Bateman gradients

**Figure 2.**
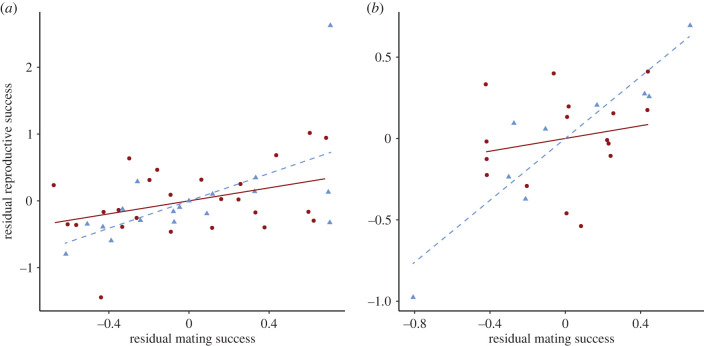
Bateman gradients estimated using residual regression in a monoecious and a dioecious population of *Sagittaria latifolia*. Residuals were calculated from the multiple regression of the number of ramets and the average size of each ramet (measured as average mid-vein length) per MLL onto reproductive success and mating success per MLL via its female or male function. Fitted Bateman gradients are shown for female (solid red lines) and male (dashed blue lines) function in each population. Dots represent the residual reproductive success and residual mating success for each MLL per population (using circles for female function and triangles for male function). (*a*) In the monoecious population, these values represent the residual reproductive and mating success of MLLs via their female and their male sex functions. (*b*) In the dioecious population, these values are the residual reproductive and mating success of female or male MLLs. (Online version in colour.)

In the monoecious population, there was a significant, positive association between mating and reproductive success for male function (*β*_mm_ = 1.02 ± 0.35 s.e.; table 1, figure 2*a*; electronic supplementary material, table S8). For female function, this association was weaker and non-significant (*β*_ff_ = 0.42 ± 0.26 s.e.). As a result, the sex difference in Bateman gradients was positive in the monoecious population (*Δβ*_ss_ = 0.60 ± 0.42 s.e.; electronic supplementary material, table S9). Patterns of sexual selection were similar in the dioecious population, where there was a significant positive gradient for males (*β*_m_ = 0.95 ± 0.15 s.e.) and a weaker, non-significant gradient for females (*β*_f_ = 0.20 ± 0.25 s.e.; table 1, figure 2*b*; electronic supplementary material, table S8). These sex-specific values of the Bateman gradient yielded a positive value of the sex difference in Bateman gradients in the dioecious population (Δ*β*_ss_ = 0.76 ± 0.30 s.e.; table 1). Alternative calculations of Bateman gradients that accounted for variation in plant size (multiple regression and bootstrapping) yielded qualitatively similar results, with evidence for stronger sexual selection in males (or male function) than in females (or female function; electronic supplementary material). By contrast, calculations of Bateman gradients that did not account for variation in plant size yielded larger values of *β* for female function than for male function (monoecious population: *β*_ff_ = 1.20 ± 0.30 s.e., *β*_mm_ =1.17 ± 0.10 s.e.; dioecious population: *β*_f_ = 2.16 ± 0.42 s.e., *β*_m_ = 0.88 ± 0.09 s.e.; electronic supplementary material, table S3) leading to negative values of *Δβ*_ss_ in both populations (monoecious population: Δ*β*_ss_ = −0.03 ± 0.30 s.e.; dioecious population: Δ*β*_ss_ = −1.28 ± 0.45 s.e.; electronic supplementary material, table S4).

## 4. Discussion

Our data reveal strong sexual selection operating on male function in natural populations of *S. latifolia*. Patterns of sexual selection were similar in the monoecious and dioecious populations studied here and might reflect the fact that—at the inflorescence level—plants in monoecious populations are temporally unisexual, limiting the kinds of interference that might otherwise restrict the magnitude of sexual selection in hermaphrodites. Our analysis indicated that sexual selection did not operate on female function in either population. However, total mating and reproductive success were size-dependent, with similar patterns of size dependency for both female and male function in each population. On one hand, these patterns underscore the size dependency of instantaneous measures of fitness in modular organisms [59]. On the other, because size variation in natural populations is probably driven by non-heritable differences in plant age and local growing conditions [60], these data also highlight the insights that can be gained by accounting for variation in plant size when considering forms of selection that affect one phase of the life cycle. Below, we discuss our findings and interpretations in more detail and consider their implications for life-history evolution in *S. latifolia*.

In the two natural populations of *S. latifolia* studied here, sexual selection occurred via male but not female function. Similar patterns have been observed for dioecious *Mercurialis annua* [16] and are consistent with the results of studies of selection on floral traits that might affect mating success via male function in plants (e.g. selection on anther dimorphism [61,62] or petal size [63,64]). Indeed, a previous study involving mating arrays of *S. latifolia* found evidence for sexual selection on male function that operated via variation in floral display size (the number and size of open flowers [23]). In general, pollinators prefer to visit larger floral displays [65–67], but for members of the genus *Sagittaria*, which is characterized by plants with unisexual flowers, a positive association between display size and pollinator visitation appears to apply to male but not female sex functions [42] (and see [68]). For female function, the association between display size and pollinator visitation is weaker, and females in dioecious populations are not mate (pollen)-limited [42], indicating that any associations between display size and reproductive success are driven by resource—instead of mate-limitation. Moreover, for congeneric *Sagittaria trifolia*, greater pollinator visitation is associated with increased pollen removal from male flowers but independent of pollen receipt by female flowers [12]. Together, these patterns of pollinator visitation provide a mechanistic explanation for the operation of sexual selection in *S. latifolia*, with variation in floral display size driving variation in mating success for males (and male function in monoecious populations) but not females (and female function in monoecious populations).

Using non-size-corrected data would have yielded steeper Bateman gradients for female than for male function in both populations and negative Δ*β*_ss_ values, a result that would have been consistent with sexual selection on female function. Indeed, our estimates of *I*_f_, the magnitude of the opportunity for selection via female function, were comparable to those from a study reporting sexual selection on the female function of hermaphroditic *Passiflora incarnata* [35]. However, in both this study and the study of *Passiflora*, size was a major predictor of total reproductive success via female function. For the *Passiflora* study, size variation was driven by the application of different levels of a nutrient fertilizer treatment. For *S. latifolia*, size variation is expected to emerge via differences in clone age [69] and local growing conditions [50]. Variation resulting from differences in age and local conditions are largely non-heritable [70] and the need to correct for plant size when measuring selection on reproductive traits has been recognized in previous studies [71].

Our estimates of Bateman gradients in monoecious and dioecious populations indicated that sexual selection operated similarly in both populations. The magnitude of sexual selection can depend on the extent to which sex roles are separated during mating [26,39]. When mating via female and male function occurs simultaneously, mating success for male and female sex functions may covary. As a result, any cross-sex effects that arise from that covariance weaken the sex-specific dependence of reproductive success on mating success, reducing the scope for sexual selection to operate on mating success via male roles without also affecting mating success via female roles (and *vice versa*). For example, the freshwater snail *Biomphalaria glabrata* typically engages in reciprocal mating via the alternation of sex roles during mating encounters, and the estimation of cross-sex effects indicates that selection on mating success is sexually mutualistic (mating benefits both sex roles; [26]). As a result, reproductive success via male function depends not only on the mating success of snails via their male function, but also on their mating success via female function [26]. Conversely, for hermaphroditic organisms that deploy their sex functions at different times (e.g. individuals are temporally unisexual), mating via male and female roles involves separate (and at least partly independent) mating events, reducing the magnitude of cross-sex effects [26,72]. This is the case for *S. latifolia*—inflorescences are functionally unisexual when they are visited by pollinators. Thus, there should be reduced the scope for covariance in mating success between sex roles, something that could be confirmed in a study focussed on estimating cross-sex effects in monoecious *S. latifolia*.

The magnitude of sexual selection is influenced by the amount of time each sex is available for mating, in part via effects on operational sex ratios [73]. All else being equal, selection on the frequency of mating events is stronger on the sex that spends less time out of the mating pool (i.e. the sex with the shorter ‘dry’ time [73]). For dioecious *S. latifolia*, males produce more flowers and open those flowers more gradually than females [74], yielding strongly male-biased operational (floral) sex ratios during peak flowering ([74], this study) and a shorter ‘dry time’ for males compared to females. Short dry times for males should be associated with selection for high mating frequency (i.e. a positive Bateman gradient [73]) and favour plants with greater investment in traits that promote mating success via male function [73]. Flower size is strongly sexually dimorphic in dioecious populations of *S. latifolia*, and there is a positive association between pollinator visitation and the degree of flower size dimorphism between females and males [42]. Total floral display size (the number and size of flowers) has previously been shown to be subject to sexual selection in mating arrays comprised of male and hermaphrodite plants [23]. Our study of a natural population of dioecious *S. latifolia* complements these previous findings by revealing a positive Bateman gradient for males.

Floral sex ratios were less strongly biased in the monoecious population studied here—they were nearly 50 : 50—but does this mean that sexual selection should necessarily be a weaker force in monoecious than in dioecious populations of *S. latifolia* where floral sex ratios were male-biased? As long as male fitness is solely determined by mating rate, the answer should be ‘no’ [73]. Two features of *S. latifolia* should make mating rate the primary driver of fitness via male function, in spite of unbiased floral sex ratios. First, and most obviously, the positive Bateman gradient in the monoecious population studied here indicates that mating success drives reproductive fitness through male function. Second, life-history trade-offs arising from investment in male function appear to be weaker than those for the investment in female function [47]. In particular, whether there are any negative fitness consequences for the production of male flowers may be context-dependent [75]. Investment in female function by *S. latifolia* plants has strong and direct consequences for plant (clonal) growth and survival [45]. Although there appear to be some costs associated with the production of male flowers (via reductions in total plant nitrogen content [47]), in natural populations, these costs do not appear to directly affect measures of plant growth (including total size [45]), perhaps because the costs associated with male investment can be recovered more easily than those associated with female investment, at least in some environments [75]. Together, a positive Bateman gradient and weaker life-history trade-offs for male function should favour selection on traits that increase male mating success.

Measurements of the magnitude of sexual selection in natural populations of plants are rare—certainly in comparison with the numbers of studies of animal populations. Here we found a positive association between mating and reproductive success for males in a dioecious population and for the male function of hermaphrodites in a monoecious population of *S. latifolia*. By contrast, and after accounting for the effects of plant size on fecundity, mating success was not associated with reproductive success for female function. Accordingly, sexual selection appeared to operate on male but not female function in the two populations studied here. Should similar results also apply to other plants? In general, the answer seems to be ‘yes’ [2,21,39,55], but whether that answer applies to any particular plant depends on the processes generating variation in mating success. For *S. latifolia*, there is not only considerable variance in mating success; there is also a positive, linear association between investment in male function and siring success [23,74]. However, this kind of association is not thought to apply to most plants, which are usually considered to be subject to a decelerating association between investment in male function and reproductive fitness (i.e. there is a saturating male gain curve [21,76]). Specifically, increased investment in pollen production is not expected to yield commensurate increases to mating or reproductive success, in part because plant immobility and localized pollinator foraging should generally result in diminishing returns on investment in male function via local mate competition [77]. Indeed, local mate competition is expected to have strong effects on the strength of sexual selection [78,79]. Moreover, many plants are thought to be pollen-limited [80], which could lead to sexual selection on female function via positive associations between mating and reproductive success [35]. Thus, although sexual selection can operate in plant populations, many more studies from natural populations will be required before any general statements about the importance of sexual selection in plants can be made.

## Data Availability

Data and R code for the analyses are available from the Dryad Digital Repository: https://doi.org/10.5061/dryad.1c59zw3z1 [81]. Supplementary material is available online [82].
